# Local Infiltrative Analgesia of Murine Femur Fractures In Vivo Does Not Inhibit Fracture Healing

**DOI:** 10.7759/cureus.23569

**Published:** 2022-03-28

**Authors:** Andrew F Tyler, Jaimo Ahn, Derek J Donegan

**Affiliations:** 1 Orthopaedic Surgery, Hospital Corporation of America (HCA) Houston Healthcare Clear Lake, Webster, USA; 2 Orthopaedic Surgery, University of Michigan, Ann Arbor, USA; 3 Orthopaedic Surgery, University of Pennsylvania, Philadelphia, USA

**Keywords:** animal model, trauma, pain management, opioid, femur fracture, local anesthetic

## Abstract

The opioid epidemic in the United States has forced care providers to seek out alternatives to narcotic analgesics. Physicians involved in trauma care, including orthopaedic trauma surgeons, often have patients requiring significant amounts of these medications, especially in the perioperative period, given the acuity and severity of their injuries. Modalities such as local infiltration of fractures with anesthetic agents during operative treatment may provide some benefit to this population by decreasing postoperative pain and narcotic usage. However, prior data suggest that these agents are chondrotoxic, which may impede secondary fracture healing. The purpose of this study was to investigate the hypothesis that local anesthetics decrease secondary bone healing and callus formation in stabilized murine femur fractures through chondrocyte apoptosis. Male C57BL/6 mice underwent intramedullary stabilization and fracture of bilateral femurs followed by immediate infiltration of the fracture site with local anesthetic agents. Femurs were dissected at 10- and 20-days post-fracture and evaluated by $\mu$CT and histological analysis. No significant differences were seen in callus size or mineralization between controls and fractures treated with a local anesthetic. When the callus was analyzed histologically, local anesthetic agents appeared to increase cartilage density. Therefore, infiltration of local anesthetics during operative treatment of fracture as part of a multimodal approach to pain control does not appear to significantly affect callus formation in a preclinical model, although subclinical molecular effects may be present. Local infiltrative analgesia with local anesthetics may be used as an adjunct for perioperative pain control during femur fracture surgery without a significant effect on secondary bone healing.

## Introduction

With more than 116 million Americans living with chronic pain and over 90% of these patients receiving opioids for the management of their symptoms, opioid use and abuse have developed into a national epidemic with significant health and economic repercussions [1]. While these substances are sometimes purchased illicitly, they are more often obtained as prescription medications from physicians and other medical care providers. Unfortunately, orthopaedic surgeons have contributed to the magnitude of the problem. Although they make up only 2.3% of physicians in the United States, they are the third-highest prescribers of opioids [2]. In addition, they treat a highly susceptible patient population: orthopaedic trauma patients are at particular risk of opioid abuse as they are at higher risk of using opioids before the injury, a finding associated with longer postoperative use and one which can lead to more psychological distress, worse symptoms, and more disability when compared with other types of patients [3].

Interest in reducing the number of opioid medications prescribed to surgical patients has led to innovations in the treatment of perioperative pain. A recent study investigating local infiltrative analgesia (LIA) of a cocktail containing local anesthetic demonstrated reduced pain scores and decreased total opioid consumption in the acute postoperative period after operative treatment of femur fractures without significant complications [4]. However, the effects of local anesthetic on fracture healing are unknown. Studies on cultured chondrocytes suggest that local anesthetics lead to apoptosis and cellular death [5-7]. As secondary bone healing depends on the viability of cartilaginous tissue for effective bone healing, this suggests that infiltration of the fracture site with local anesthetics may inhibit fracture healing and lead to delayed union or nonunion. Prior animal studies from the 1960s indicated this did not occur but utilized relatively insensitive methodology and did not include newer compounds developed since that time [8,9].

This study aimed to evaluate the effect of local anesthetic on secondary bone formation using a traumatic murine long-bone fracture model, hypothesizing that local anesthetic would decrease secondary bone formation within the fracture callus. The findings of this study would further clarify the safety of LIA in perioperative pain management during operative treatment of fractures.

## Materials and methods

Animal model

The Institutional Animal Care and Use Committee (IACUC) of the University of Pennsylvania approved all protocols related to the use of rodents in this study (protocol #806493). Male C57BL/6 mice (8-10 weeks old) (n = 4 per study arm, 20 total animals) underwent intramedullary femoral stabilization with a 25-gauge hypodermic needle followed by bilateral diaphyseal femur fracture using a three-point bending fracture apparatus under isoflurane anesthesia as previously described [10]. Mice were sedated with isoflurane (4%-5% for induction, 1%-2% for maintenance) until they were not responsive to hind limb pinch and maintained a respiratory rate of 55-100 breaths per minute. The knee joint was removed of hair from the mid-tibia to the mid-femur using electric clippers. The pin site was cleaned using 70% ethanol and povidone-iodine, allowed to dry, then draped to isolate the knee. The 25-gauge needle was then inserted percutaneously through the distal end of the femur and advanced until a hard stop was noted at the proximal femur. If the femur was not appropriately cannulated as noted by palpation of the needle tip in the subcutaneous tissues, the procedure was repeated until successful. This protocol was then repeated for the contralateral extremity. Once both limbs were cannulated, bilateral femurs were fractured using the custom three-point bending apparatus, which had been previously calibrated to create transverse midshaft femur fractures in C57BL/6 mice. Immediately after fracture, each animal received an injection at the fracture site of local anesthetic (experimental) or phosphate-buffered saline (PBS) of equal volume (control). The following quantities of local anesthetic were used in equipotent amounts based on literature values: lidocaine (10 mg/mL [APP Pharmaceuticals, Fresenius, Germany]) (1000 mg/injection), ropivacaine (5 mg/mL [Akorn, Illinois, US]) (500 mg/injection), bupivacaine (2.5 mg/mL [Hospira, Illinois, US]) (400 mg/injection), liposomal bupivacaine (13.3 mg/mL bupivacaine, [Exparel, Pacira Pharmaceuticals, California, US]) (400 mg/injection) [11,12]. The amount of ropivacaine was based on the Koehler study and was adjusted for murine physiology [13]. Mice were injected with buprenorphine SR Lab (ZooPharm, Wyoming, US) at a dose of 1.0 mg/kg subcutaneously immediately following fracture for pain control and monitored according to IACUC guidelines, including 15 minutes evaluating recovery from anesthesia and over the ensuing 72 hours to confirm that the animals were able to ambulate. Animals were euthanized via carbon dioxide gas chamber followed by cervical dislocation 10- or 20- days post-fracture for analysis. No experimental or control animals were excluded from the study. Experimental and control animals were kept in mixed cages to reduce confounding variables. All experiments were performed by the lead author (AT).

Tissue harvesting and processing

Bilateral femurs were dissected from the surrounding soft tissue, and the intramedullary pins were removed. Femurs were then fixed in 4% paraformaldehyde at 4°C for 72 hours, washed with 70% ethanol, then transferred to 50 mL centrifuge tubes (Falcon) packed with moistening gauze for mCT evaluation. Following CT analysis, femurs were washed in PBS, then decalcified for 48 hours at room temperature in 10 mL of Immunocal (StatLab Medical Products, Dallas, US), then dehydrated in 70% ethanol. Samples were embedded in paraffin then sectioned into 5 mM thick slices along the coronal plane of the femur and placed on glass slides for histologic analyses.

$\mu$CT analysis

A vivaCT 40 $\mu$CT scanner (Scanco Medical, Switzerland) was used for analysis. Femurs were loaded into 30 mm diameter tubes then scanned at 55 kVp and 145 mA with 1000 slices per 180° using an isotropic three-dimensional voxel size of 10 mm, a 200 ms integration time, and a fixed global threshold corresponding to a mineral density of 220 mg hydroxyapatite [HA]/cm^3^ as the maximum gray value applied to distinguish mineralized from unmineralized tissue. A semiautomated process was used to identify fracture callus and exclude cortical bone as previously described [14]. Briefly, callus and cortical bone sections were identified manually with spline interpolation performed every 10 slices with points reviewed with re-interpolation as necessary. Cortical bone sections were then removed manually using a similar process followed by calculation of the following parameters: total volume, bone volume, BV/TV, bone mineral density, and tissue mineral density.

Histology

Bone and callus tissue analysis was performed using sections stained with Safranin O and Fast Green for brightfield microscopy analysis of collagen as previously described [14]. An Eclipse Ni-E motorized upright microscope (Nikon Instruments, Tokyo) was used for analysis. ImageJ (NIH) was used for image evaluation.

Statistical analysis

*A priori* power analysis was performed for $\alpha$ = .05 and 1-$\beta$ = 0.8 to evaluate for a 25% difference in $\mu$CT parameters based on previously reported values and sample sizes in the literature for total volume and bone volume in C57BL/6 mice [15]. A two-way analysis of variance (ANOVA) was used with post hoc multiple comparisons to the control group (PBS) using Dunnett’s test. A value of *p* < .05 was considered to be significant. All statistics were performed using GraphPad Prism 8 (GraphPad Software, California, US).

## Results

**Figure 1:**
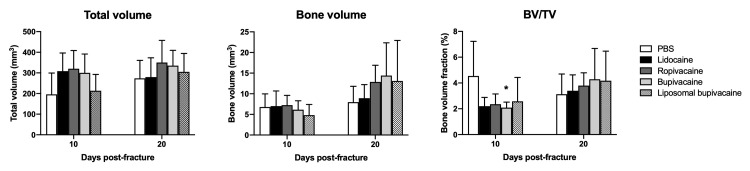
$\mu$ CT analysis of total volume, bone volume, and bone volume fraction of callus formation of fractured mouse femurs after treatment with local anesthetic * = *p* < .05

Secondary bone healing was measured by quantifying the extent of mineralized callus formation via $\mu$CT analysis. The total volume, bone volume, and bone volume fraction of femur fracture callus 10 and 20 days after fracture were compared between control mice (PBS) and those that received local anesthetic injections of lidocaine, ropivacaine, bupivacaine, and liposomal bupivacaine (Figure 1). While there was a slight difference between controls and fractures treated with bupivacaine at day 10 post-fracture (*p* = .04), there were no other statistically significant differences noted between the groups at either time point.

**Figure 2:**
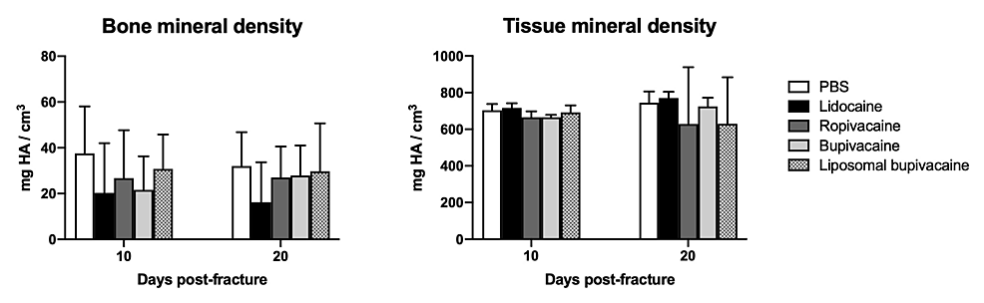
$\mu$ CT analysis of callus mineralization of fractured mouse femurs after treatment with local anesthetic

As a secondary measure, mineralization of the callus was also assessed. Bone and tissue mineral density were compared at 10 and 20 days after fracture between the control and experimental cohorts (Figure 2), again via $\mu$CT analysis. There were no statistically significant differences noted between any of the groups at either time point.

**Figure 3:**
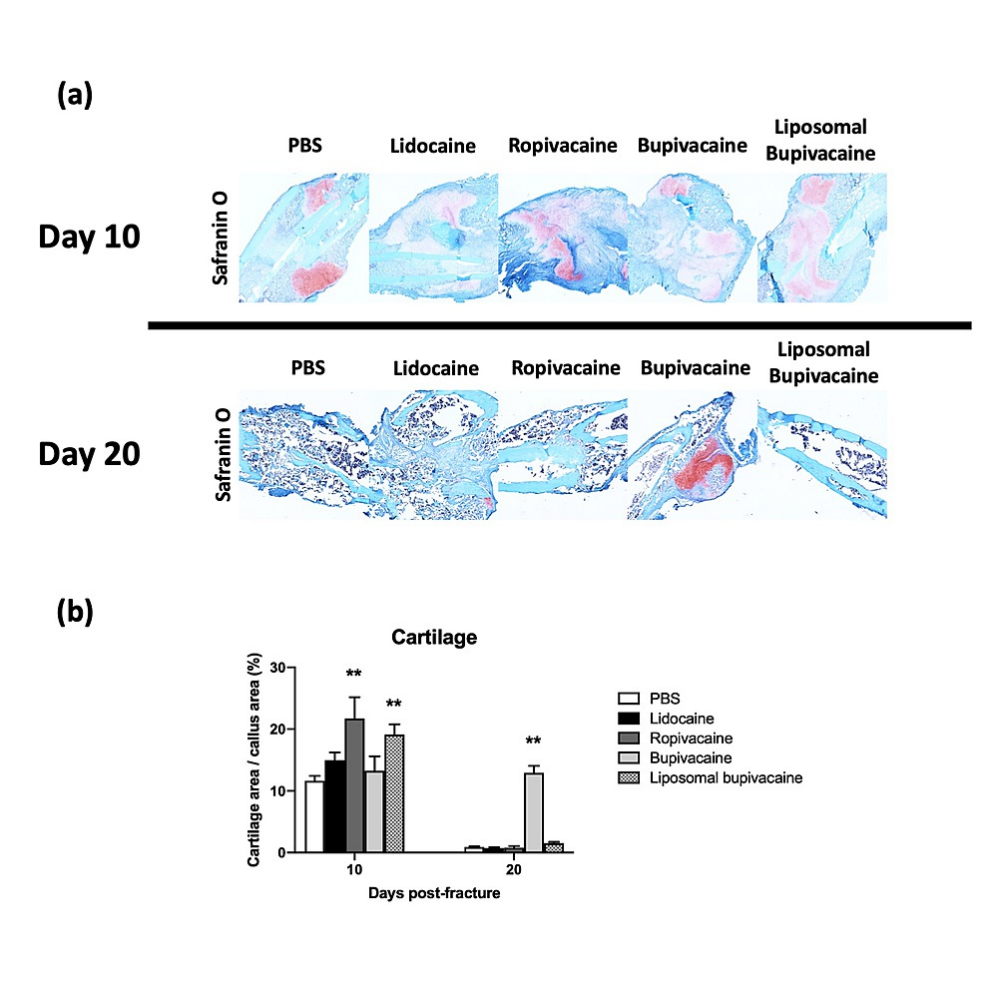
Cartilage staining of fracture callus after treatment with local anesthetic (A) Qualitative histology using cartilage-specific (Safranin O) staining. (B) Quantification of the cartilage as a ratio to the fracture callus. * = *p* < .05, ** = *p* < 0.01.

After $\mu$CT analysis, samples were stained with a cartilage-specific stain (Safranin O) as described in the methods (Figure 3A). The ratio of cartilage to callus was evaluated at 10 and 20 days after fracture using a color threshold analysis available on the ImageJ software suite. Fractures treated with ropivacaine or liposomal bupivacaine showed increased cartilage density 10 days after a fracture that normalized at 20 days when compared to controls. Fractures treated with bupivacaine did not demonstrate a difference in cartilage density 10 days after fracture but appeared to maintain a significant fraction of cartilage 20 days after fracture (Figure 3B). Fractures treated with lidocaine did not show any significant difference when compared to controls.

## Discussion

Prescription opioid use in the United States has reached epidemic proportions. In 2017, the opioid crisis was declared a national public health emergency [16]. Over 47,000 deaths were attributed to opioid overdose that year, with prescription opioid pain relievers accounting for over a third of these deaths [17]. While public health measures such as improved access to antidotes such as naloxone are critical, medical care providers continue to seek out alternatives to narcotics in the treatment of both acute and chronic pain.

In 2017, Koehler and colleagues demonstrated that surgical site injection of a multimodal analgesic cocktail containing the local anesthetic ropivacaine in patients undergoing operative treatment of femur fractures led to decreased pain scores and less total narcotic consumption in the acute postoperative period without any significant perioperative complications [4]. Local infiltration analgesia (LIA) has been used for adjuvant pain control in other orthopaedic procedures (including those involving cartilage) with mixed results. However, studies have also demonstrated that local anesthetics have a deleterious effect on chondrocyte viability. Grishko et al. exposed cultured primary human chondrocytes to lidocaine, ropivacaine, and bupivacaine and demonstrated that these compounds cause mitochondrial dysfunction and apoptosis in these cell types [6]. A similar effect was shown by Piper et al. after treatment of full-thickness cartilage explants and cultures chondrocytes treated with ropivacaine and bupivacaine [7].

Clinically, the phenomenon of cartilage damage due to local anesthetic exposure has been most strongly associated with the glenohumeral joint after arthroscopic shoulder surgery. While no direct causal link has been made, a correlation between post-arthroscopic glenohumeral chondrolysis (PAGCL) and the use of local anesthetic infusions has been demonstrated in several studies [18,19]. Most notable of these is that of Wiater et al. where 395 cases were evaluated retrospectively; of the 49 cases identified to have chondrolysis in the 18 months following surgery, all were found to have received an intraarticular infusion of local anesthetic (lidocaine or bupivacaine) after the index procedure [20]. Other studies have shown a similar, if not as impressive, effect. However, these findings have not been reproduced in procedures involving other joints such as the knee or hip.

Additionally, preclinical models have failed to demonstrate a deleterious effect of local anesthetic infiltration on cartilage viability. Gothman showed that rabbit tibial fractures treated with intramedullary nailing and local infiltration of lidocaine showed earlier callus formation and increased vascular infiltration when compared to controls [9]. Conversely, Flatmark similarly treated rabbit radial fractures and found no such differences [8]. Similarly, Henry et al. demonstrated that hematoma block with lidocaine or bupivacaine of rat femur fractures did not affect fracture callus force to failure, mean DNA content, or mean collagen content when compared to controls [21]. Nevertheless, the methodology used in each of these studies was meant to analyze gross tissue changes and lacks the precision of more contemporary techniques.

The current study aimed to further evaluate the preclinical model in light of the novel clinical application of LIA in fracture surgery. As secondary bone healing progresses through a cartilaginous intermediate (i.e., callus), the hypothesis was that LIA using potential chondrotoxic such as local anesthetic would decrease the size and quality of callus. By employing the treatment strategy used in studies of human femoral fracture followed by *ex vivo* analysis using $\mu$CT, any significant differences, even if quantitatively small, in callus formation could be ascertained at a microscopic level.

In regard to the callus architecture and mineralization as evaluated by $\mu$CT, no major significant differences were seen between any of the groups. There were no statistically significant differences noted in total volume (TV), bone volume (BV), bone mineral density (BMD), or tissue mineral density (TMD) between femur fractures treated with lidocaine, ropivacaine, or bupivacaine in equipotent amounts when compared with controls. Moreover, there were no significant differences noted in the same variables in femur fractures treated with an equipotent amount of liposomal bupivacaine, a formulation that extends the half-life of the compound *in vivo*. While there was a slight decrease in bone volume fraction (BV/TV) in animals treated with bupivacaine 10 days after fracture, this difference appeared to resolve at 20 days.

However, on a more cellular level, small but interesting differences appear between the groups. The longer-acting anesthetics, including ropivacaine, bupivacaine, and liposomal bupivacaine, appeared to increase the amount of cartilage seen in the fracture callus at the earlier time point. This may be due to improved analgesia leading to increased weight-bearing and movement, which in turn may stimulate secondary bone healing in accordance with Wolff’s law. However, this difference did not appear to significantly influence the bony architecture that developed as the fractures healed and therefore may be clinically irrelevant. These data suggest that LIA with local anesthetics may be a safe technique for reducing opioid consumption and pain in the postoperative period after surgical fracture treatment; however, it should continue to be explored further given the cellular effects noted in this study.

There are several potential weaknesses with this study. As no significant differences were found in the CT data, the conclusions are vulnerable to a type 2 error, and small differences in the microarchitecture may be present even if undetected. However, the likelihood that such small differences would have a clinically meaningful effect on the ultimate biomechanical strength of the bone is low. Furthermore, the treatment protocol of a single injection at the time of fracture may have been insufficient to induce observable changes. However, this mirrors the protocol used in the human clinical study and is, therefore, less relevant to the question at hand. Lastly, the timing of analysis may have been inappropriate to evaluate differences in callus formation, and earlier time points may have shown a greater effect on chondrocyte viability. That said, the time points chosen to reflect those demonstrated in the literature to be accurate representations of early and late callus were used accordingly.

## Conclusions

This study demonstrates that local infiltrative analgesia using local anesthetics, including liposomal formulations, does not significantly, affect final callus formation after femur fracture in an animal model. Therefore, it is likely that these modalities are safe to use adjuncts for perioperative pain control in patients undergoing operative treatment of long bone fractures.
